# Dermal delivery of amitriptyline for topical analgesia

**DOI:** 10.1007/s13346-021-00982-x

**Published:** 2021-04-22

**Authors:** Chin-Ping Kung, Bruno C. Sil, Yanling Zhang, Jonathan Hadgraft, Majella E. Lane, Bhumik Patel, Renée McCulloch

**Affiliations:** 1 UCL School of Pharmacy, 29-39 Brunswick Square, London, WC1N 1AX UK; 2 London Metropolitan University, 166-220 Holloway Road, London, N7 8DB UK; 3 Great Ormond Street Hospital for Children, Great Ormond Street, London, WC1N 3JH UK

**Keywords:** Amitriptyline, Skin permeation, Penetration enhancement, Topical analgesic, Co-solvent system

## Abstract

**Abstract:**

Amitriptyline, administered orally, is currently one of the treatment options for the management of neuropathic pain and migraine. Because of the physicochemical properties of the molecule, amitriptyline is also a promising candidate for delivery as a topical analgesic. Here we report the dermal delivery of amitriptyline from a range of simple formulations. The first stage of the work required the conversion of amitriptyline hydrochloride to the free base form as confirmed by nuclear magnetic resonance (NMR). Distribution coefficient values were measured at pH 6, 6.5, 7, and 7.4. Solubility and stability of amitriptyline were assessed prior to conducting in vitro permeation and mass balance studies. The compound demonstrated instability in phosphate-buffered saline (PBS) dependent on pH. Volatile formulations comprising of isopropyl alcohol (IPA) and isopropyl myristate (IPM) or propylene glycol (PG) were evaluated in porcine skin under finite dose conditions. Compared with neat IPM, the IPM:IPA vehicles promoted 8-fold and 5-fold increases in the amount of amitriptyline that permeated at 24 h. Formulations containing PG also appear to be promising vehicles for dermal delivery of amitriptyline, typically delivering higher amounts of amitriptyline than the IPM:IPA vehicles. The results reported here suggest that further optimization of topical amitriptyline formulations should be pursued towards development of a product for clinical investigational studies.

**Graphical abstract:**

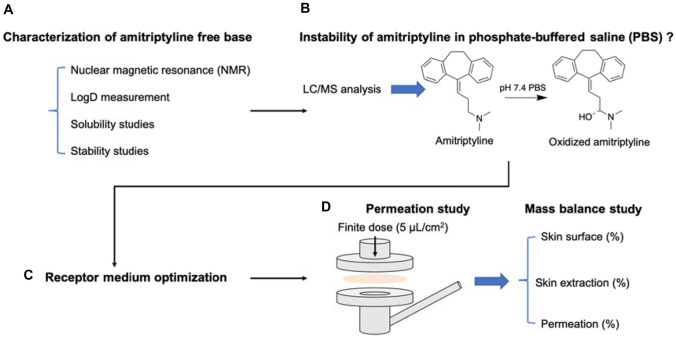

**Supplementary Information:**

The online version contains supplementary material available at 10.1007/s13346-021-00982-x.

## Introduction

Amitriptyline is a dibenzocycloheptene-derivative tricyclic antidepressant (TCA) which also has potent analgesic effects. Oral amitriptyline is currently used to treat neuropathic pain [1–3]. Amitriptyline inhibits norepinephrine and serotonin reuptake [4] and blocks many peripheral receptors, including alpha adrenergic, muscarinic, histaminergic, nicotinic, and NMDA receptors [5–8]. The antinociceptive mechanism of action of amitriptyline also involves adenosine and opioid receptors [9, 10]. In addition, in vitro and in vivo studies show that amitriptyline is a more potent voltage-gated sodium channel blocker in peripheral nerves with a longer duration of analgesia compared with bupivacaine [11, 12]. The combination of these actions may contribute to the peripheral efficacy of amitriptyline [13, 14].

Topical amitriptyline at various concentrations has been studied in clinical trials, and its efficacy has been reviewed by Casale et al. [15]. Some controlled clinical trials have also evaluated the combination of amitriptyline with other drugs in topical formulations. The combination of topical amitriptyline and ketamine is one of the most popular investigational treatments for neuropathic pain. AmiKet™ (EpiCept Corporation, USA) is a cream containing amitriptyline 4% and ketamine 2%. It has completed phase 1 and 2 clinical trials. The outcomes of three phase 2 clinical trials involving 945 patients with post-herpetic neuralgia or diabetic painful neuropathy were reviewed in detail by Sawynok and Zinger [16]. The analgesic effect was reported as concentration-related. These trials showed the efficacy of AmiKet (4% amitriptyline plus 2% ketamine) for patients with post-herpetic neuralgia and diabetic painful neuropathy. Barton et al. [17] also reported a double-blind, randomized, placebo-controlled trial enrolling 203 patients with chemotherapy-induced peripheral neuropathy. Patients were instructed to apply 1.31 g pluronic lecithin organogel gel containing 10 mg baclofen, 40 mg amitriptyline hydrochloride, and 20 mg ketamine hydrochloride to the area of pain. The results suggested a good improvement in both the sensory subscale (*p* = 0.053) and motor neuropathy subscale (*p* = 0.021) and a slight improvement in the autonomic subscale (*p* = 0.580) compared with a placebo.

Although topical amitriptyline has been widely studied in clinical trials, there is limited information in the literature concerning the skin absorption of amitriptyline. Stott et al. [18] investigated the in vitro permeation of amitriptyline in human skin using complex coacervation as a penetration enhancement method. The skin flux from an amitriptyline: sodium deoxycholate coacervate was reported as 6.6 ± 0.71 μg/cm^2^/h, which was 2.2-fold higher than the flux from a control solution. However, the authors did not disclose the formulation doses applied in the study. More recently, Liu et al. [19] conducted in vitro permeation studies in porcine skin under infinite dose conditions (0.5 mL/0.785 cm^2^) to evaluate the permeation of amitriptyline from a 15 mM amitriptyline suspension in a 20% propylene glycol (PG)/pH 7.4 buffer vehicle. The formulation delivered 14.27 ± 1.56% of amitriptyline through porcine skin after 24 h.

Volatile formulations have previously been explored as a strategy to enhance skin delivery of actives. Oliveira et al. [20] reported the effects of ethanol on methyl paraben permeation across silicone membrane and human skin under finite dose conditions. The presence of ethanol in Transcutol P® (TC), dimethyl isosorbide, and isopropyl myristate (IPM) formulations led to increases in the percentage of applied methyl paraben that permeated through human skin. Santos et al. [21] reported finite dose permeation studies for fentanyl in human skin. Formulations containing IPM and ethanol at different degrees of drug saturation (DS) were tested, and results confirmed that increasing DS promoted fentanyl permeation. IPM is one of the most commonly used fatty acid esters in dermal preparations. PG, a safe and effective solvent for dermal delivery, is also widely used in topical and transdermal formulations. Additionally, the efficacy of PG for delivery of analgesics such as fentanyl and methadone has been reported previously [22, 23]. Therefore, in the present work, we aimed to investigate skin delivery of amitriptyline from formulations comprising isopropyl alcohol (IPA) combined with PG or IPM. A concentration of 4% w/v amitriptyline base solutions was chosen because this is within the range of 1–10% for topical amitriptyline formulations that were previously used in clinical studies [15, 24]. The concentration is also in line with AmiKet™ (4% amitriptyline plus 2% ketamine) [16]. A number of other solvents commonly used in dermal delivery were also evaluated for their compatibility with amitriptyline. Studies were conducted under finite dose conditions in porcine skin. As the unionized form of a drug (free acid or free base) is generally preferable for topical and transdermal delivery [25, 26], the free base form of the drug was used for permeation studies.

## Materials and methods

### Materials

Amitriptyline hydrochloride (AMI-HCl), Brij™ O20, dichloromethane (DCM), anhydrous acetone, and sodium carbonate were obtained from Sigma-Aldrich (UK). Sodium dihydrogen phosphate, sodium hydrogen phosphate, magnesium sulphate, and dithiothreitol (DTT) were supplied by Fisher Scientific (UK). Phosphate-buffered saline (pH 7.3 ± 0.2 at 25 °C) was prepared using Dulbecco A tablets supplied by Oxoid (UK). Octyl salicylate (OSAL), PG, IPM, IPA, and 1-octanol were obtained from Sigma-Aldrich (UK). High-performance liquid chromatography (HPLC) grade water, HPLC grade acetonitrile, HPLC grade methanol, liquid chromatography/mass spectrometry (LC-MS) grade acetonitrile, LC-MS grade 0.1% trifluoroacetic acid (TFA) solution, limonene (LIM), ethyl acetate (EtAc), and TFA were supplied by Fisher Scientific (UK). Chloroform-d (CDCl_3_) were obtained from Cambridge Isotope Laboratories Inc. (UK). Propylene glycol monolaurate (PGML) Type II and TC were gifts from Gattefossé (St. Priest, France). All chemicals, unless otherwise stated, were ≥ 99% purity.

### Preparation of amitriptyline base

The method for the preparation of amitriptyline free base was developed in-house. About 30 mL of HPLC grade water was added to dissolve 3.14 g of amitriptyline hydrochloride (0.01 mol). The pH of the reaction solution was adjusted to 9–10 with 0.5 mol/L sodium carbonate solution to convert amitriptyline hydrochloride to amitriptyline free base. About 50 mL of dichloromethane was then added to extract amitriptyline free base. The reaction solution was allowed to equilibrate by stirring at 25 °C for 60 min. Subsequently, the organic layer was separated and the aqueous layer washed three times with dichloromethane (3 × 50 mL). The organic layers were collected together and dried with magnesium sulphate. Dichloromethane was removed by using a rotary evaporator (Heidolph, UK) and placed on a high vacuum line for five hours. Amitriptyline free base (2.65 g) was obtained as a thick oil with a yield of 95 ± 2%. Molecular characterization was performed by proton nuclear magnetic resonance (^1^H NMR) spectroscopy to confirm the structure. The spectrum was obtained in chloroform-*d* using a Bruker Avance 500 MHz NMR spectrometer (Bruker Corporation, USA) and processed using MestReNova 11.0.4 (Mestrelab Research, Spain). The NMR spectral signals are as follows: ^1^H NMR (500 MHz, Chloroform-d) *δ* 7.34–7.30 (m, 1H), 7.26–7.13 (m, 7H), 7.08–7.05 (m, 1H), 5.91 (t, 1H), 3.52–3.22 (m, 2H), 2.90 (d, 2H), 2.46–2.28 (m, 4H), 2.20 (s, 6H).

### HPLC and LC-MS analysis

The HPLC system used in this study consisted of an Agilent G1322A degasser, G1311A quaternary pump, G1313A auto sampler, G1316A thermostat column compartment, and G1315B diode array detector (Agilent Technologies, USA). The software used to acquire and analyse the data was ChemStation® for LC 3D, Rev. A. 09.03 (Agilent Technologies, USA). Analysis was performed with a Luna® Omega 5 μm PS C_18_ 150 × 4.60 mm column (Phenomenex, UK) equipped with a universal HPLC guard column (Phenomenex, UK) packed with a SecurityGuard™ C_18_ cartridge (Phenomenex, UK). For the analysis of amitriptyline, acetonitrile and 0.1% TFA aqueous solution were selected as the mobile phase and gradient conditions were applied. The concentration of acetonitrile increased from 30 to 75% over 14 min. The mobile phase was then returned to the initial proportion (30:70 0.1% TFA: acetonitrile) within 0.5 min and hold for 5 min to ensure system equilibrium before next injection. A flow rate of 1 mL/min was used. The column temperature and injection volume were set to 25 °C and 10 μL, respectively. The wavelength used for the analysis of amitriptyline was 238 nm.

LC-MS was used to identify the degradation products of amitriptyline during the stability studies. The LC-MS system used in the study consisted of an Agilent G1322A degasser, G7111B quaternary pump, G7129A auto sampler, G1316A thermostat column compartment G7114A VWD detector coupled with G6135C single quadrupole mass spectrometer (Agilent Technologies, UK). The software used to acquire and analyse the data was OpenLAB CDS ChemStation® software (Agilent Technologies, UK). The analysis was performed using a Luna® Omega 5 μm PS C_18_ 150 × 4.60 mm column (Phenomenex, UK) equipped with a universal HPLC guard column (Phenomenex, UK) packed with a SecurityGuard™ C18 cartridge (Phenomenex, UK). An ion-spray voltage of 10 kV was applied, and the desolvation temperature was set to 350 °C. Nitrogen was used as the curtain gas (35 psi) and collision gas. The spectra were scanned in the positive ion peak over the m/z range of 100–750. For the chromatographic conditions, the mobile phase consisted of LC-MS grade 0.1% TFA aqueous solution and acetonitrile. The concentration of acetonitrile increased from 30 to 75% over 16 min and then returned to starting conditions within 0.5 min. The mobile phase proportion was kept at 30:70 0.1% TFA: acetonitrile for 5 min before next injection. A flow rate of 1 mL/min was used. The column temperature and injection volume were set to 25 °C and 10 μL, respectively. The wavelength used for UV detection was 238 nm.

### Solubility parameter, solubility, and distribution coefficient determination

The solubility parameters (*δ*) of several solvents were calculated by means of the van Krevelen and Hoftyzer type 3-D approach [27] using Molecular Modelling Pro® Version 6.3.3 software (ChemSW, Fairfield, CA, USA). For solubility measurements, an excess amount of amitriptyline free base or amitriptyline hydrochloride was added to 0.5 mL of each solvent in Eppendorf® tubes (*n* = 3). These Eppendorf® tubes were sealed with Parafilm® and placed on a rotator at 32 ± 2 °C for at least 48 h. These samples were then centrifuged at 12,000 rpm for 15 min at 32 ± 1 °C. The supernatant was suitably diluted with methanol and analysed using the validated HPLC method (method validation parameters are shown in Table S1).

The method used for logarithm of distribution coefficient (Log D) measurement was the shake flask method, adapted from published OECD guidelines [28]. About 0.2 M sodium phosphate buffer solutions at pH = 6, 6.5, 7, and 7.4 were prepared and mutually saturated with 1-octanol by slow-stirring (at 130 rpm) for 48 h at 25 °C. The solutions were equilibrated in a separating funnel for 24 h before separation. Amitriptyline solutions at 0.1 and 0.5 mmol/L were prepared in the 1-octanol phase. The solutions were mixed with the aqueous phase (sodium phosphate buffers) at three different ratios (1:1, 2:1, and 1:2) in Eppendorf® tubes (*n* = 3). To reach equilibrium, these Eppendorf® tubes were rotated gently approximately one hundred times through 180° at room temperature, allowing the trapped air to rise through the two phases. Before sampling, tubes were then centrifuged at room temperature and at 13,000 rpm for 30 min so that the two phases separated from each other. Samples were taken from each phase with suitable dilution using methanol before HPLC analysis.

### Stability studies

The chemical stability studies of amitriptyline in neat solvents and in the Franz cell receptor medium were conducted for 96 h. A known quantity of amitriptyline was dissolved in the receptor medium or neat solvent. These solutions were placed in Eppendorf® tubes and sealed with Parafilm® before being placed in a shaker (VWR, UK) at 32 ± 1 °C. Samples were taken at 0, 24, 48, 72, and 96 h. The samples were diluted with methanol where necessary and analysed using HPLC or LC-MS.

### Preparation of porcine skin

Porcine tissue was obtained from a local abattoir and prepared on the day of slaughter. The porcine ear skin was separated from cartilage at room temperature. Firstly, porcine tissue was washed with distilled water to remove dirt from the skin surface. Next, the superficial hair was trimmed with special care to avoid damage to the skin barrier. The skin was then separated slowly with the help of a scalpel and tweezers. After separation, the membrane was washed with distilled water and excess water was removed using tissue paper. Finally, the porcine tissue was stored in the freezer (− 30 °C) until required. Before a permeation study, frozen porcine skin was rapidly cut into discs using a cork-borer. The membrane was cleaned with HPLC grade water and blotted dry with filter paper before use.

### In vitro permeation studies

In vitro permeation studies in full-thickness porcine skin were conducted according to OECD guideline no. 428 [29]. The skin integrity was assessed by measuring impedance at a frequency of 50 Hz before applying formulations [30]. The skin was considered intact if the impedance reading was above 15 kΩ at 50 Hz. Diffusion cells with an impedance value < 15 kΩ were discarded. Custom-made vertical glass Franz diffusion cells with a diffusion area of 1 cm^2^ were used. The assembled Franz cells were filled with 2.2 mL of degassed receptor medium (PBS pH 7.4 with 6% (w/v) Brij™ O20 and 0.05% (w/v) dithiothreitol). About 7-mm Teflon®-coated magnetic stir bars were then added into the receptor compartment, and all Franz cells were left in a thermostatted water bath at 34.5 ± 1 °C (SUB Aqua 26 Plus, Grant, UK) for at least 30 min with stirring to ensure the membrane surface temperature was 32 ± 1 °C. After the equilibration period, a finite dose (5 μL/cm^2^) of 4% w/v amitriptyline formulation was applied evenly to the membrane surface. 200-μL aliquots were withdrawn from the receptor compartment and replaced with fresh receptor medium at 0, 2, 4, 6, 8, 10, 12, and 24 h. All samples were analysed to quantify the amount of amitriptyline permeated using the validated HPLC method.

### Mass balance studies

Mass balance studies were performed after each permeation study. The skin surface was washed three times with 1 mL of methanol. The skin was then placed in Eppendorf® tubes with 1 mL of methanol. The samples for skin extraction were placed in a shaker overnight to extract active ingredients. All Eppendorf ® tubes were centrifuged at 13,000 rpm at 32 °C for 15 min before sampling. The supernatant was sampled and diluted with methanol where necessary and analysed by HPLC.

### Statistical analysis

Microsoft Excel® 2016 (Microsoft Corporation, USA) was used for data processing and the calculation of mean and standard deviation (SD). IBM® SPSS Statistics® 24.0 was used for statistical analysis. The Shapiro–Wilk test was used to assess the normality of the data. If the *p* value calculated from the test was higher than 0.05, the data were assumed to be normally distributed. For normally distributed data (parametric data), the independent-samples *t* test and one-way analysis of variance (ANOVA) with Tukey’s HSD post hoc test were used to analyse 2 groups and ≥ 3 groups respectively. For non-normally distributed data (non-parametric data), the Mann–Whitney *U* test was used to test statistical significance between two groups and the Kruskal–Wallis one-way ANOVA test was performed to investigate statistical differences among different groups (≥ 3 groups). A probability of *p* < 0.05 was considered as a statistically significant difference.

## Results and discussion

### Preparation and characterization of amitriptyline free base

Successful conversion of amitriptyline hydrochloride to the free base was confirmed by ^1^H NMR (Fig. S1). A peak at 5.3 ppm was assigned to dichloromethane, the residual solvent. The Log *p* value of amitriptyline was reported as 4.92 by Hansch et al. [31]. However, the experimental Log *D* values at pH 6–7.4 have not been reported. Therefore, Log *D* values of amitriptyline were measured at different pH values, and the results are listed in Table 1. The Log *D* value of amitriptyline increased with increasing pH values. The Log *D* value obtained at pH 7.4 for amitriptyline was 2.28 ± 0.02. The predicted log *D* value at pH 7.4 from ACD/Labs is 2.96.

**Table 1:** Distribution coefficient of amitriptyline base at different pH values

pH	Log D*
6.00	1.36 ± 0.01
6.50	1.64 ± 0.02
7.00	2.07 ± 0.03
7.40	2.28 ± 0.02

*Mean ± SD (*n* = 18)

### Solubility and stability of amitriptyline

The solubility of amitriptyline free base and its hydrochloride salt in several common solvents for dermal delivery were assessed and the results are summarized in Table S2. Amitriptyline shows excellent solubility not only in hydrophobic solvents but also in some hydrophilic solvents such as PG and TC. As expected, amitriptyline hydrochloride showed high solubility only in polar solvents such as PG and TC.

**Fig. 1:**
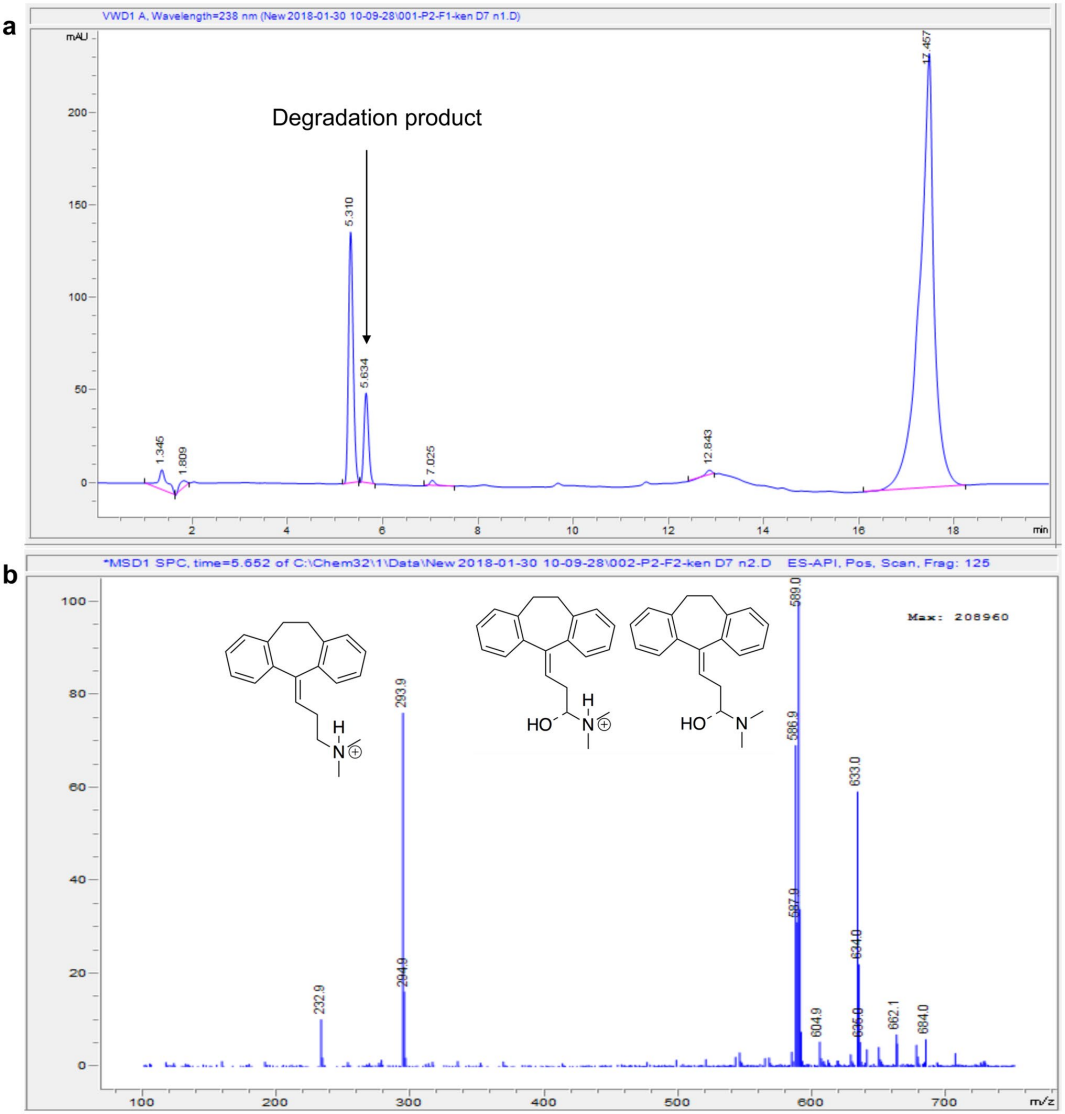
LC-MS analysis of amitriptyline hydrochloride in pH 7.4 PBS + 6% Brij™ O20 at 32 ± 1 °C after 96 h. **(a)** A degradation product peak of amitriptline was observed. **(b)** The mass spectrum of the degradation product

To quantify accurately the amount of amitriptyline, the chemical stability of amitriptyline in the receptor medium was assessed. Therefore, stability studies for amitriptyline free base were conducted in the receptor medium. About 6% w/v Brij™ O20 was added to the receptor medium to ensure sink conditions were maintained for amitriptyline during in vitro permeation studies (solubility of amitriptyline base in 6% w/v Brij™ O20 was 14.15 ± 0.04 mg/mL at 32 ± 1 °C). During the stability studies of amitriptyline in pH 7.4 PBS (with 6% Brij™ O20), an unknown peak appeared, and the area of this peak increased with time, while the area of the peak of amitriptyline decreased with time (Fig. 1; Table 2). The recovery of amitriptyline in PBS with or without Brij™ O20 was ~ 75% at 24 h.

**Table 2:** Recovery (%) of amitriptyline hydrochloride in pH 7.4 PBS and 6% Brij™ O20 in pH 7.4 PBS after 24, 48, 72, and 96 h at 32 ± 1 °C (*n* = 3; mean ± SD)

Solvents	6% Brij™ O20 in pH 7.4 PBS	pH 7.4 PBS
*T* = 0 h	100.0	100.0
*T* = 24 h	75.5 ± 0.7	75.2 ± 0.6
*T* = 48 h	72.1 ± 0.7	71.4 ± 0.6
*T* = 72 h	71.5 ± 1.1	63.4 ± 0.2
*T* = 96 h	65.9 ± 0.8	57.5 ± 0.3

To investigate further the degradation of amitriptyline in PBS, LC-MS was used to analyse the amitriptyline degradation product. This peak at 5.6 min is assigned to the amitriptyline degradation product which eluted slightly later than amitriptyline (Fig. 1). Mass spectrometry data suggest that the degradation product is likely to be a dimer of oxidized amitriptyline (m/z 586.9 for [M + H]^+^). Although the single oxidized amitriptyline monomer (m/z 293.9 for [M + H]^+^) is expected to be more polar than amitriptyline, the dimer of oxidized amitriptyline might be expected to be more hydrophobic. This is consistent with the slightly longer retention time of the oxidation product of amitriptyline compared with amitriptyline.

**Fig. 2:**
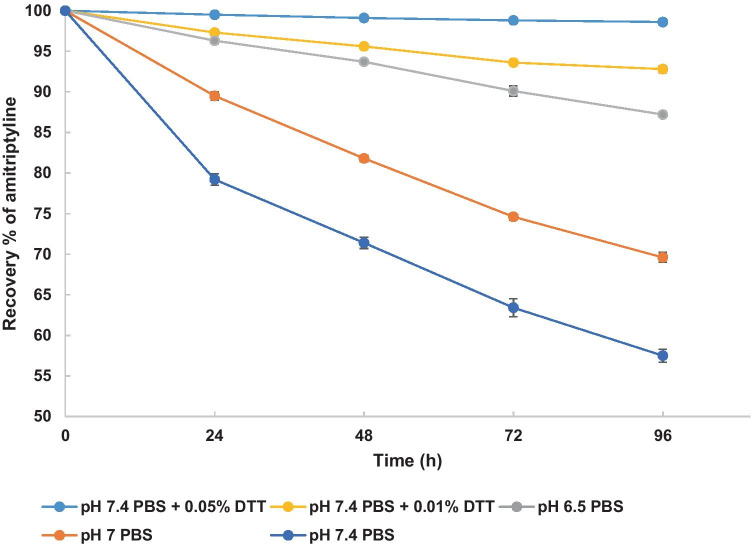
Recovery of amitriptyline hydrochloride in different receptor phases after 24, 48, 72, and 96 h at 32 ± 1 °C (*n* = 3; mean ± SD). The recovery of amitriptyline hydrochloride in pH 7.4 PBS with 0.05% DTT was higher than 98.5% over 96 h

To reduce the loss of amitriptyline in the receptor medium, one approach would be to add an antioxidant. Different concentrations of DTT (0.1%, 0.05%) were therefore added to 6% Brij™ O20 pH 7.4 PBS to examine stabilization of amitriptyline. The effect of pH on the oxidation process of amitriptyline was also evaluated during the stability studies. The results are shown in Fig. 2. The oxidation rate of amitriptyline in PBS was observed to be pH dependent following the order pH 6.5 < pH 7.0 < pH 7.4. Amitriptyline is stable over 96 h after addition of 0.05% (w/v) DTT to pH 7.4 PBS with 6% (w/v) Brij™ O20. Therefore, when 0.05% (w/v) DTT is added to the receptor medium, it should reduce the amount of oxidized amitriptyline during permeation studies compared with studies conducted with no antioxidant in the receptor medium. In addition, no adverse effect on skin barrier was detected by impedance assessment when 0.05% (w/v) DTT and 6% (w/v) Brij™ O20 were added to the receptor medium (Fig. S2).

**Fig. 3:**
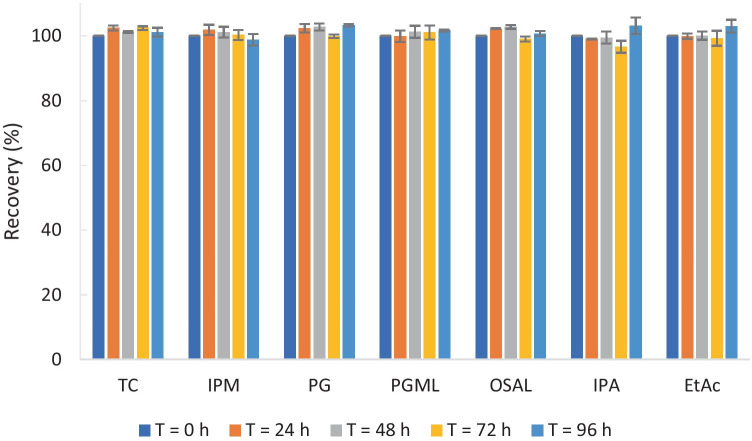
Recovery (%) of amitriptyline free base in a series of neat solvents after 24, 48, 72, and 96 h at 32 ± 1 °C (*n* = 3; mean ± SD). TC Transcutol®P, IPM isopropyl myristate, PG propylene glycol, PGML propylene glycol monolaurate, OSAL octyl salicylate, IPA isopropyl alcohol, EtAc ethyl acetate

The stability of amitriptyline free base in neat TC, IPM, PG, PGML, OSAL, IPA, and EtAc was also assessed. The results are shown in Fig. 3. The recovery values in all these solvents were above 95%. The chemical stability of amitriptyline free base in these solvents at 32 ± 1 °C (over 96 h) was therefore confirmed. IPM and PG which show contrasting physicochemical properties and favourable solubility of amitriptyline free base (Table S2) were selected as the model solvents take forward in combination with IPA.

### The influence of IPM/IPA binary systems

**Fig. 4:**
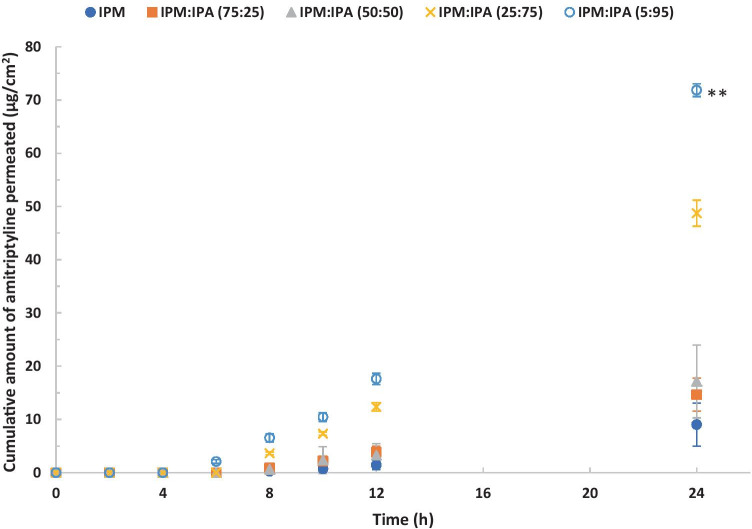
Permeation profiles of amitriptyline in porcine ear skin after application of 4% (w/v) amitriptyline base in IPM (blue circle), IPM:IPA (75:25) (orange square), IPM:IPA (50:50) (gray triangle), IPM:IPA (25:75) (yellow cross sign), and IPM:IPA (5:95) (light blue) for finite dose (5 μL/cm^2^) conditions at 32 ± 1 °C (mean ± SD; 4 ≤ *n* ≤ 5). IPM isopropyl myristate, IPA isopropyl alcohol. Statistical differences were obtained using one-way ANOVA followed by Tukey’s multiple comparisons test where appropriate, *p < 0.05, **p < 0.01

IPM is commonly used in pharmaceutical and cosmetic formulations [32]. IPA is a volatile solvent with a vapour pressure value of 4.4 kPa at 20 °C [33]. It has also been used in a number of topical products, and it is considered safe for application to the skin [34]. The effects of the combined use of IPM and IPA on amitriptyline permeation were studied under finite dose conditions. The permeation profiles of amitriptyline from IPM/IPA binary formulations are shown in Fig. 4. The highest cumulative amount of amitriptyline that permeated through porcine skin at 24 h was observed for IPM:IPA (5:95) (one-way ANOVA, *p* < 0.01), followed by IPM:IPA (25:75). There were 8.0-fold and 5.4-fold increases in the amount of amitriptyline that permeated at 24 h for IPM:IPA (5:95) and IPM:IPA (25:75), respectively, compared with neat IPM. This indicates that the high concentration of IPA in IPM/IPA binary formulations contribute greatly to an increased skin permeation. The increase in thermodynamic activity of amitriptyline base after the rapid evaporation of IPA may facilitate the partition of amitriptyline from IPM into the *stratum corneum*.

**Fig. 5:**
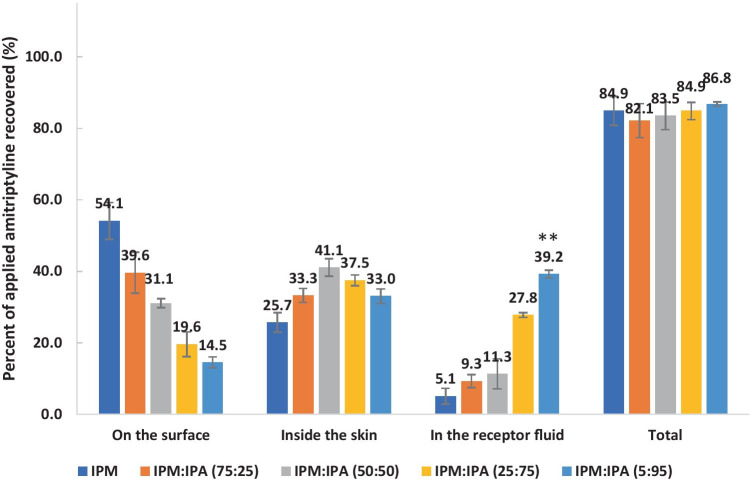
Percentages of amitriptyline recovered from the skin surface, skin extraction, and following skin permeation after 24-h permeation studies in porcine skin. Finite doses (5 μL/cm^2^) of 4% (w/v) amitriptyline base in IPM, IPM:IPA (75:25), IPM:IPA (50:50), IPM:IPA (25:75), and IPM:IPA (5:95) were applied in permeation studies (mean ± SD; 4 ≤ *n* ≤ 5). IPM isopropyl myristate, IPA isopropyl alcohol. Statistical differences were obtained using one-way ANOVA followed by Tukey’s multiple comparisons test where appropriate, *p < 0.05, **p < 0.01

The mass balance results for the IPM/IPA binary systems are shown in Fig. 5. For the IPM/IPA binary systems 33.0–41.1% of the applied amitriptyline dose was recovered from the skin. This indicates that skin may serve as a reservoir for amitriptyline. The hydrophilic nature of the dermal layer can also be a barrier for the diffusion of amitriptyline, a highly lipophilic compound [35]. The permeation of amitriptyline increased with the concentration of IPA in IPM/IPA binary formulations, while the percentage of amitriptyline remaining on the skin surface decreased with the increasing concentration of IPA. This indicates that the presence of IPA plays a key role in enhancing the skin permeation of amitriptyline. The IPM:IPA (5:95) formulation delivered a significantly higher (one-way ANOVA, *p* < 0.01) percentage of applied amitriptyline through porcine skin compared with the other IPM/IPA binary formulations. Only 14.52 ± 1.53% of applied amitriptyline from the IPM:IPA (5:95) formulation remained on the skin surface after 24 h. Therefore, IPM:IPA (5:95) may be a promising formulation for the delivery of amitriptyline base.

Sato et al. [36] suggested that IPM may affect the diffusivity of actives in the *stratum corneum* and increase the partition of both drugs and solvents from vehicles into the *stratum corneum*. The integration of IPM with lipids of the *stratum corneum* may fluidize the lamellar-gel phase of the lipids [37]. Oliveira et al. [38] conducted methyl paraben permeation studies and evaluated the human *stratum corneum* uptake of solvents, including IPM. The authors reported that the highest partition coefficient of methyl paraben was obtained from IPM compared with other solvents, including dimethyl isosorbide, Transcutol®, polyethylene glycol 200, and polyethylene glycol 400. Haque et al. [39] evaluated the distribution of a number of solvents for finite dose permeation studies of anthramycin. The results showed that IPM did not permeate through skin while ~ 10% of IPM was recovered following skin extraction.

The influence of PG/IPA binary systems.

**Fig. 6:**
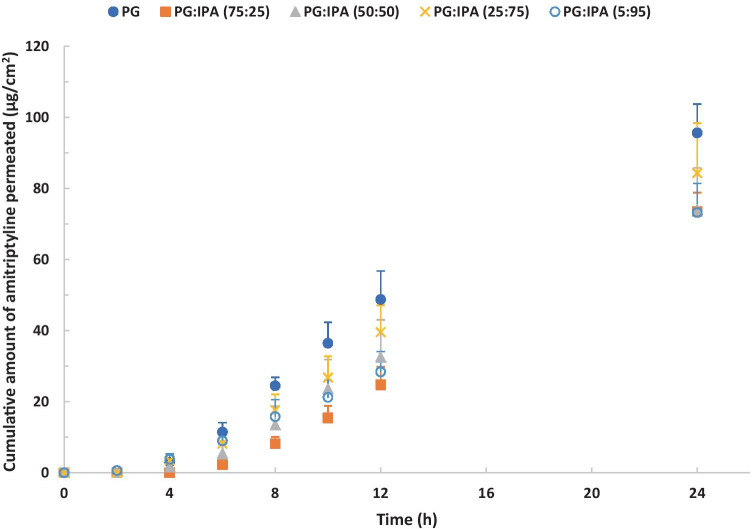
Permeation profiles of amitriptyline in porcine ear skin after application of 4% (w/v) amitriptyline base in PG (blue circle), PG:IPA (75:25) (orange square), PG:IPA (50:50) (gray triangle), PG:IPA (25:75) (yellow cross sign), and PG:IPA (5:95) (light blue circle) for finite dose (5 μL/cm^2^) conditions at 32 ± 1 °C (mean ± SD; 4 ≤ *n* ≤ 5). IPM isopropyl myristate, IPA isopropyl alcohol. Statistical differences were obtained using one-way ANOVA followed by Tukey’s multiple comparisons test where appropriate, *p < 0.05, **p < 0.01

The permeation profiles of amitriptyline for PG/IPA binary formulations are shown in Fig. 6. Although there were no significant differences (one-way ANOVA, *p* > 0.05) between the cumulative amounts of amitriptyline that permeated from PG/IPA systems at 24 h, all formulations delivered more than 70 μg/cm^2^ of amitriptyline through porcine skin. Unlike the IPM/IPA systems, the permeation of amitriptyline from PG/IPA systems did not increase with the concentration of IPA in the applied formulations.

**Fig. 7:**
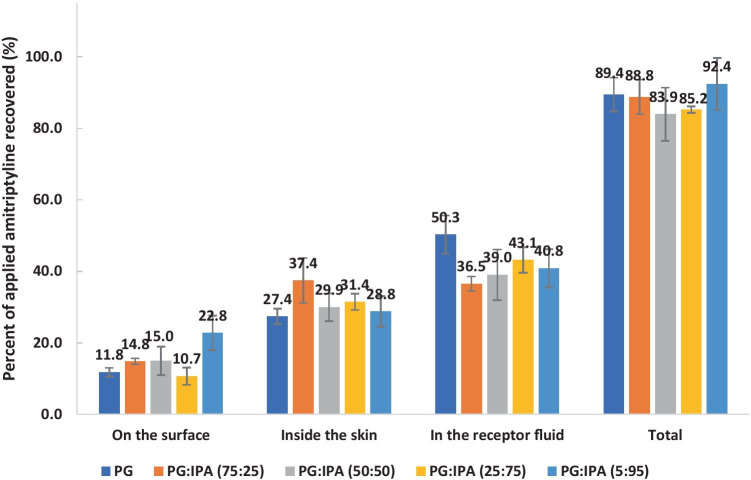
Percentages of amitriptyline recovered from the skin surface, skin extraction, and following skin permeation after 24-h permeation studies in porcine skin. Finite doses (5 μL/cm^2^) of 4% (w/v) amitriptyline base in PG, PG:IPA (75:25), PG:IPA (50:50), PG:IPA (25:75), and PG:IPA (5:95) were applied in permeation studies (mean ± SD; 4 ≤ *n* ≤ 5). IPM isopropyl myristate, IPA isopropyl alcohol. Statistical differences were obtained using one-way ANOVA followed by Tukey’s multiple comparisons test where appropriate, *p < 0.05, **p < 0.01

The results of the mass balance studies for the PG/IPA systems are shown in Fig. 7. In general, the highest percentages of applied amitriptyline were recovered from the receptor medium, followed by skin extraction, and from the skin surface. Only 11.8–22.8% of the applied amitriptyline remained on the skin surface at 24 h. This indicates that all PG/IPA binary formulations are promising vehicles for dermal delivery of amitriptyline base. The overall recoveries for all the formulations tested in the present study were > 80%.

In general, PG and PG/IPA binary systems delivered higher amounts of amitriptyline to the skin compared with IPM/IPA binary systems. Amitriptyline base is a thick oil at room temperature with a molecular weight of 277 Da. The solubility parameter of amitriptyline base is 10.54 (cal/cm^3^)^1/2^ which is close to the reported solubility parameters of *stratum corneum* lipids (9.7–10 (cal/cm^3^)^1/2^) [40]. This indicates amitriptyline base should have favourable solubility in *stratum corneum* lipids. Amitriptyline base has high solubility (> 1000 mg/mL) in PG at 32 ± 1 °C (Table S2). The uptake of PG into skin may increase further the solubility of amitriptyline in the skin and promote the partition of amitriptyline into the skin. A number of studies have investigated the effects of PG on skin permeation of actives [41–51]. The skin permeation of both metronidazole and PG from various formulations containing PG have been reported. The results showed that the permeation of metronidazole increased with the degree of PG permeation. Therefore, the authors suggested that PG may enhance metronidazole permeation by acting as a “carrier solvent” [49]. Kasting et al. [45] conducted in vitro permeation studies in human skin under infinite dose conditions (150 μL per 0.79 cm^2^). The results showed that PG enhanced the skin permeation of triprolidine base compared with lipophilic solvents such as IPM and mineral oil. The authors suggested that PG was incorporated into the *stratum corneum* and modified the solubility of the permeant in *stratum corneum*. Trottet et al. [47] reported the skin permeation of loperamide and PG in human skin. Loperamide hydrochloride formulations with 12–40% PG were assessed under finite dose conditions (10 mg/cm^2^). The results showed that the fluxes of both loperamide and PG through human skin were proportional to the applied doses of propylene glycol. More recently, Haque et al. [39] conducted finite dose permeation studies of anthramycin in human skin and reported the skin uptake of vehicles such as PG and TC. The authors confirmed that the majority of PG applied permeated through the skin rather than remaining on the skin surface.

## Conclusion

Amitriptyline is currently given orally for the management of neuropathic pain and migraine. The molecule has favourable propeties for demal delivery but few studies have examined the skin permeation of amitriptyline. The present work demonstrated effective skin penetration of amitriptyline from a range of vehicles based on IPA, PG and IPM. For permeation studies, the instability of amitriptyline in PBS was overcome by inclusion of a suitable additive, namely, 0.05% (w/v) DTT. The maximum cumulative amounts of the drug that permeated were obtained with PG, PG/IPA binary systems and IPM:IPA (5:95), without significant differences among these systems. About 72–96 μg/cm^2^ of amitriptyline can be delivered through skin at 24 h under finite dose conditions. Future work will focus on further optimization of these preparations and evaluation in human skin permeation studies with a view to progressing them towards development in the clinic.

## Supplementary Information

Below is the link to the electronic supplementary material.

Supplementary file1 (DOCX 141 KB)

## Data Availability

The authors confirm that the data supporting the findings of this study are available within the article and supplementary materials. Raw data are available from the corresponding author on request.

## References

[1] Gilron I, Watson CPN, Cahill CM, Moulin DE (2006). Neuropathic pain: a practical guide for the clinician. Can Med Assoc J.

[2] O'Connor AB, Dworkin RH (2009). Treatment of neuropathic pain: an overview of recent guidelines. Am J Med.

[3] Attal N, Cruccu G, Baron Ra, Haanpää M, Hansson P, Jensen TS, et al. EFNS guidelines on the pharmacological treatment of neuropathic pain: 2010 revision. Eur J Neurol. 2010;17(9):1113. 10.1111/j.1468-1331.2010.02999.x 20402746

[4] Sánchez C, Hyttel J (1999). Comparison of the effects of antidepressants and their metabolites on reuptake of biogenic amines and on receptor binding. Cell Mol Neurobiol.

[5] Traiffort E, Pollard H, Moreau J, Ruat M, Schwartz J, Martinez-Mir M (1992). Pharmacological characterization and autoradiographic localization of histamine H2 receptors in human brain identified with [125I] iodoaminopotentidine. J Neurochem.

[6] Eisenach J, Gebhart G (1995). Intrathecal amitriptyline acts as an N-methyl-D-aspartate receptor antagonist in the presence of inflammatory hyperalgesia in rats. Anesthesiology.

[7] Park TJ, Shin SY, Suh BC, Suh EK, Lee IS, Kim YS (1998). Differential inhibition of catecholamine secretion by amitriptyline through blockage of nicotinic receptors, sodium channels, and calcium channels in bovine adrenal chromaffin cells. Synapse.

[8] Gray AM, Pache DM, Sewell RD (1999). Do α2-adrenoceptors play an integral role in the antinociceptive mechanism of action of antidepressant compounds?. Eur J Pharmacol.

[9] Gray A, Spencer P, Sewell R (1998). The involvement of the opioidergic system in the antinociceptive mechanism of action of antidepressant compounds. Br J Pharmacol.

[10] Sawynok J, Reid AR, Esser MJ (1999). Peripheral antinociceptive action of amitriptyline in the rat formalin test: involvement of adenosine. Pain.

[11] Gerner P, Mujtaba M, Sinnott CJ, Wang GK (2001). Amitriptyline versus bupivacaine in rat sciatic nerve blockade. Anesthesiology.

[12] Khan MA, Gerner P, Wang GK (2002). Amitriptyline for prolonged cutaneous analgesia in the rat. Anesthesiology.

[13] Sawynok J (2003). Topical and peripherally acting analgesics. Pharmacol Rev.

[14] Micó JA, Ardid D, Berrocoso E, Eschalier A (2006). Antidepressants and pain. Trends Pharmacol Sci.

[15] Casale R, Symeonidou Z, Bartolo M (2017). Topical treatments for localized neuropathic pain. Curr Pain Headache Rep.

[16] Sawynok J, Zinger C (2016). Topical amitriptyline and ketamine for post-herpetic neuralgia and other forms of neuropathic pain. Expert Opin Pharmacother.

[17] Barton DL, Wos EJ, Qin R, Mattar BI, Green NB, Lanier KS (2011). A double-blind, placebo-controlled trial of a topical treatment for chemotherapy-induced peripheral neuropathy: NCCTG trial N06CA. Support Care Cancer.

[18] Stott PW, Williams AC, Barry BW (1996). Characterization of complex coacervates of some tricyclic antidepressants and evaluation of their potential for enhancing transdermal flux. J Controlled Release.

[19] Liu K-S, Chen Y-W, Aljuffali IA, Chang C-W, Wang J-J, Fang J-Y (2016). Topically applied mesoridazine exhibits the strongest cutaneous analgesia and minimized skin disruption among tricyclic antidepressants: the skin absorption assessment. Eur J Pharm Biopharm.

[20] Oliveira G, Hadgraft J, Lane ME (2012). The influence of volatile solvents on transport across model membranes and human skin. Int J Pharm.

[21] Santos P, Watkinson A, Hadgraft J, Lane M (2012). Influence of penetration enhancer on drug permeation from volatile formulations. Int J Pharm.

[22] Carrara DNR, Grenier A, Alberti I, Henry L, Decaudin C. Transdermal delivery of systemically active central nervous system drugs. US20150005337A1; 2015.

[23] Kung C-P, Zhang Y, Sil BC, Hadgraft J, Lane ME, Patel B et al. Investigation of binary and ternary solvent systems for dermal delivery of methadone. Int J Pharm. 2020:119538. 10.1016/j.ijpharm.2020.119538 32540347

[24] Kopsky DJ, Keppel Hesselink JM (2012). High doses of topical amitriptyline in neuropathic pain: two cases and literature review. Pain Pract.

[25] Sarveiya V, Templeton JF, Benson HA (2004). Ion-pairs of ibuprofen: increased membrane diffusion. J Pharm Pharmacol.

[26] Kung C-P, Sil BC, Hadgraft J, Lane ME, Patel B, McCulloch R (2019). Preparation, Characterization and Dermal Delivery of Methadone. Pharmaceutics.

[27] van Krevelen DW. Properties of polymers: their correlation with chemical structure, their numerical estimation and prediction from additive group contributions. 3rd ed: Amsterdam: Elsevier Scientific Pub. Co. 1990;200–25.

[28] OECD. Test No. 107: Partition Coefficient (n-octanol/water): Shake flask method. Organisation for economic cooperation and development. 1995.

[29] OECD. Test No.428: Skin absorption: In vitro method. OECD guidelines for the testing of chemicals, section 4. Organisation for economic cooperation and development. 2004.

[30] Woo E, Hua P, Webster J, Tompkins W, Pallas-Areny R (1992). Skin impedance measurements using simple and compound electrodes. Med Biol Eng Comput.

[31] Hansch C, Leo A, Hoekman D, Livingstone D. Exploring QSAR: hydrophobic, electronic, and steric constants. Washington, DC: American Chemical Society; 1995.

[32] Campbell RL, Bruce RD. Comparative dermatotoxicology: I. Direct comparison of rabbit and human primary skin irritation responses to isopropylmyristate. Toxicol Appl Pharmacol. 1981;59(3):555–63. 10.1016/0041-008x(81)90310-0 7268778

[33] ILO. International Chemical Safety Cards (ICSCs). http://www.ilo.org/dyn/icsc/showcard.listcards3?p_lang=en. Accessed 30 July 2019. 2019.

[34] Heldreth B, Bergfeld WF, Belsito DV, Hill RA, Klaassen CD, Liebler D et al. Final report of the Cosmetic Ingredient Review Expert Panel on the safety assessment of methyl acetate. Int J Toxicol. 2012;31(4_suppl):112S–36S. 10.1177/1091581812444142 22869894

[35] Dick IP, Scott RC (1992). Pig ear skin as an in-vitro model for human skin permeability. J Pharm Pharmacol.

[36] Sato K, Sugibayashi K, Morimoto Y (1988). Effect and mode of action of aliphatic esters on the in vitro skin permeation of nicorandil. Int J Pharm.

[37] Leopold CS, Lippold BC (1995). An attempt to clarify the mechanism of the penetration enhancing effects of lipophilic vehicles with differential scanning calorimetry (DSC). J Pharm Pharmacol.

[38] Oliveira G, Hadgraft J, Lane M (2012). The role of vehicle interactions on permeation of an active through model membranes and human skin. Int J Cosmet Sci.

[39] Haque T, Rahman KM, Thurston DE, Hadgraft J, Lane ME. Topical delivery of anthramycin I. Influence of neat solvents. Eur J Pharm Sci. 2017;104:188–95. 10.1016/j.ejps.2017.03.043 28373034

[40] Liron Z, Cohen S (1984). Percutaneous absorption of alkanoic acids II: Application of regular solution theory. J Pharm Sci.

[41] Barrett C, Hadgraft J, Caron G, Sarkany I (1965). The effect of particle size and vehicle on the percutaneous absorption of fluocinolone acetonide. Br J Dermatol.

[42] Poulsen B, Young E, Coquilla V, Katz M (1968). Effect of topical vehicle composition on the in vitro release of fluocinolone acetonide and its acetate ester. J Pharm Sci.

[43] Coldman M, Poulsen B, Higuchi T (1969). Enhancement of percutaneous absorption by the use of volatile: nonvolatile systems as vehicles. J Pharm Sci.

[44] Davis S, Hadgraft J, Al‐Khamis K. Percutaneous absorption of methyl salicylate from polyethylene glycol vehicles. J Pharm Pharmacol. 1981;33(S1):97P-P.

[45] Kasting GB, Francis WR, Roberts GE (1993). Skin penetration enhancement of triprolidine base by propylene glycol. J Pharm Sci.

[46] Arellano A, Santoyo S, Martın C, Ygartua P (1999). Influence of propylene glycol and isopropyl myristate on the in vitro percutaneous penetration of diclofenac sodium from carbopol gels. Eur J Pharm Sci.

[47] Trottet L, Merly C, Mirza M, Hadgraft J, Davis A (2004). Effect of finite doses of propylene glycol on enhancement of in vitro percutaneous permeation of loperamide hydrochloride. Int J Pharm.

[48] Nicolazzo JA, Morgan TM, Reed BL, Finnin BC (2005). Synergistic enhancement of testosterone transdermal delivery. J Controlled Release.

[49] Hoelgaard A, Mollgaard B (1985). Dermal drug delivery—improvement by choice of vehicle or drug derivative. J Controlled Release.

[50] Watkinson R, Guy R, Hadgraft J, Lane M (2009). Optimisation of cosolvent concentration for topical drug delivery–II: influence of propylene glycol on ibuprofen permeation. Skin Pharmacol Physiol.

[51] Zhang Q, Li P, Roberts MS (2011). Maximum transepidermal flux for similar size phenolic compounds is enhanced by solvent uptake into the skin. J Controlled Release.

