# Supplementary material for: Dermal delivery of amitriptyline for topical analgesia
Source: Drug Deliv Transl Res. 2021 Apr 22;12(4):805–15. doi: 10.1007/s13346-021-00982-x (PMC8888505; doi:10.1007/s13346-021-00982-x)
Supplement: Supplementary file 1 — Supplementary file1 (DOCX 141 KB) [file 13346_2021_982_MOESM1_ESM.docx]

**Dermal delivery of amitriptyline for topical analgesia**

Chin-Ping Kung^1*^, Bruno C. Sil^2^, Yanling Zhang^1^, Jonathan Hadgraft^1^, Majella E. Lane^1^, Bhumik Patel^3^, Renée McCulloch^3^

^1^ UCL School of Pharmacy, 29-39 Brunswick Square, WC1N 1AX London, UK

^2^ London Metropolitan University, 166‐220 Holloway Road, N7 8DB London, UK

^3^ Great Ormond Street Hospital for Children, Great Ormond Street, WC1N 3JH London, UK

*** Corresponding author:** [c.kung@ucl.ac.uk](mailto:c.kung@ucl.ac.uk) (c.kung)

**Supplementary Materials**


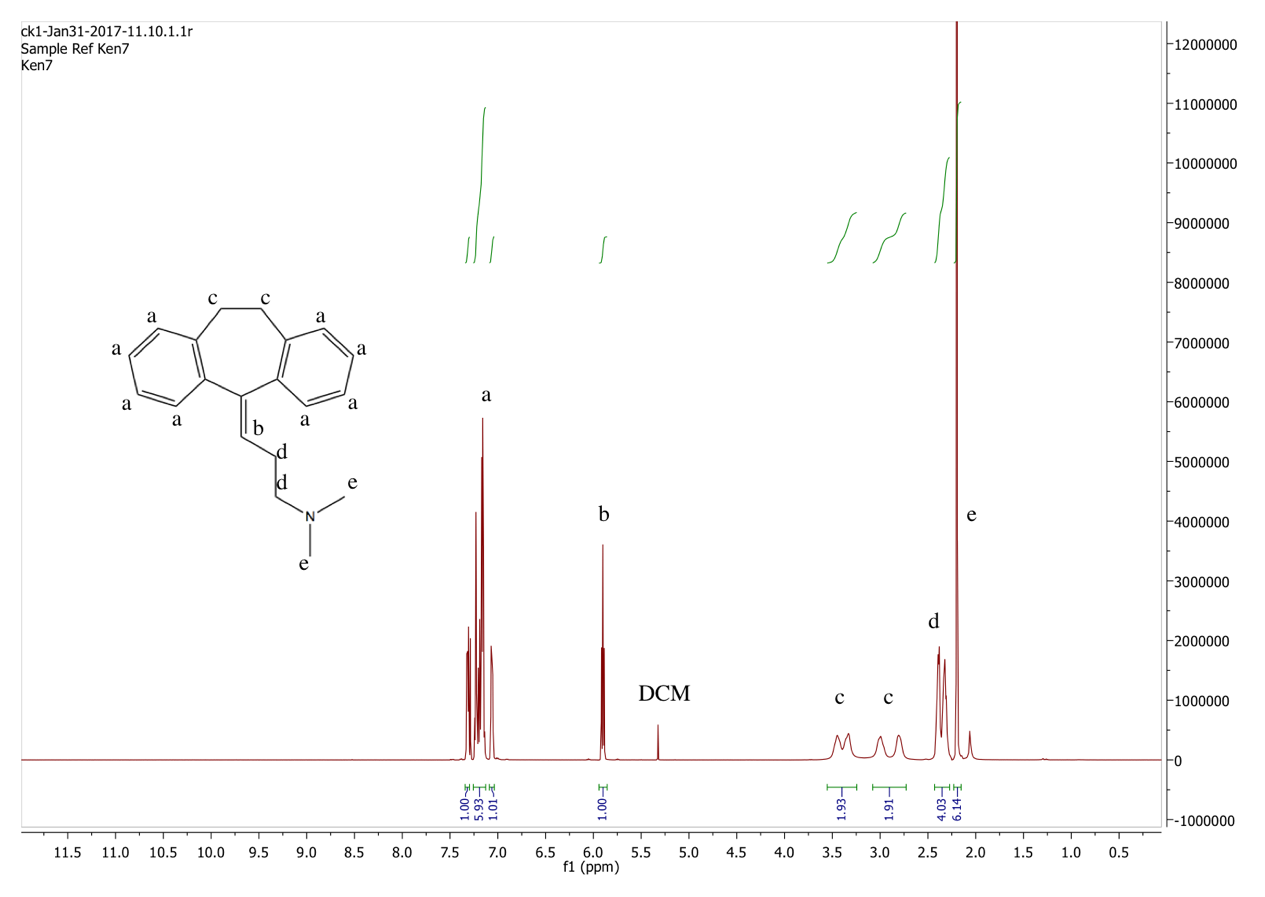


**Fig. S1.** ^1^H NMR spectrum of amitriptyline free base in chloroform-d. ^1^H NMR spectrum of amitriptyline free base in chloroform-d. ^1^H NMR (500 MHz, Chloroform-d) δ 7.34 – 7.30 (m, 1H), 7.26 – 7.13 (m, 7H), 7.08 – 7.05 (m, 1H), 5.91 (t, 1H), 3.52 – 3.22 (m, 2H), 2.90 (d, 2H), 2.46 – 2.28 (m, 4H), 2.20 (s, 6H). There is no broad singlet around 13 ppm for the hydrochloride proton. This suggests the successful conversion of amitriptyline hydrochloride to amitriptyline free base. A small peak at 5.3 ppm was assigned to dichloromethane, the residual solvent.

**Fig. S2.** Electrical impedance measured across human skin over 24 h, (n=3, mean ± SD).

**Table S1.** HPLC method validation parameters.

| **Parameters** | Concentrations (μg/mL) | **Values** |
| --- | --- | --- |
| Accuracy (%) | 0.97 | 101.61 ± 0.24 |
|  | 24.18 | 100.35 ± 0.32 |
|  | 48.36 | 100.19 ± 0.19 |
| Intra-day precision (%RSD) | 0.97 | 0.18 |
|  | 24.18 | 0.32 |
|  | 48.36 | 0.19 |
| Inter-day precision (%RSD) | 0.97 | 1.43 |
|  | 24.18 | 0.06 |
|  | 48.36 | 0.23 |
| Regression line equation |  | y = 26.96x – 3.74 |
| Range |  | 0.5 – 100 µg/mL |
| *r^2^* |  | > 0.99 |
| Limit of detection |  | 0.32 µg/mL |
| Limit of quantification |  | 0.96 µg/mL |

**Table S2.** Summary of the solubility results for amitriptyline free base and amitriptyline hydrochloride in neat solvents at 32 ± 1 °C. The calculated van Krevelen and Hoftyzer solubility parameter of amitriptyline free base is 10.54 (cal/cm^3^)^1/2^

| Solvent | Solubility parameter (cal/cm^3^)^1/2^ | Solubility of amitriptyline free base (mg/mL) | Solubility of amitriptyline hydrochloride (mg/mL) |
| --- | --- | --- | --- |
| LIM | 8.02 | > 1149.27 | 0.11 ± 0.001 |
| IPM | 8.21 | 984.49 ± 15.45 | 0.64 ± 0.09 |
| PGML | 9.44 | >1600.05 | 92.18 ± 2.13 |
| TC | 10.62 | > 1525.90 | 127.90 ± 15.80 |
| OSAL | 10.87 | 968.50 ± 10.95 | 0.11± 0.003 |
| PG | 14.06 | >1049.19 | 716.57 ± 4.13 |

LIM: limonene, IPM: isopropyl myristate, PGML: propylene glycol monolaurate, TC: Transcutol^®^P, OSAL: octyl salicylate, PG: propylene glycol.
